# Smoking and obsessive–compulsive symptoms in patients with schizophrenia, schizoaffective disorder or bipolar disorder using electronic mental health records

**DOI:** 10.1192/bjo.2025.10965

**Published:** 2026-02-16

**Authors:** Hannah R. Cohen, Chin-Kuo Chang, Deborah Ahn-Robbins, Karolina Bogdanowicz, David Chandran, Emre Kartoglu, Hitesh Shetty, Jentien M. Vermeulen, Jyoti Sanyal, Robert Stewart, Frederike Schirmbeck, Lieuwe de Haan, Richard D. Hayes

**Affiliations:** Institute of Psychiatry, Psychology, and Neuroscience, King’s College London, UK; South London and Maudsley NHS Foundation Trust, UK; Global Health Program, College of Public Health, National Taiwan University, Taipei City, Taiwan; Institute of Epidemiology and Preventive Medicine, College of Public Health, National Taiwan University, Taipei City, Taiwan; Department of Psychiatry, Amsterdam UMC, University of Amsterdam, The Netherlands; Central Institute of Mental Health, Department of Public Mental Health & Department of Psychiatry and Psychotherapy, Faculty Medicine Mannheim, Heidelberg University Mannheim, Germany; Arkin Institute for Mental Health, Amsterdam, The Netherlands

**Keywords:** Obsessive–compulsive symptom, smoking, natural language processing, severe mental illness, obsessive–compulsive disorder

## Abstract

**Background:**

Comorbid obsessive–compulsive disorder (OCD) or obsessive–compulsive symptoms (OCS) are common in people with severe mental illness (SMI; including schizophrenia, bipolar disorder and schizoaffective disorder), with little known about associations with smoking.

**Aims:**

To estimate the association between OCD/OCS and smoking status among people with SMI in a huge electronic database.

**Method:**

Using the Clinical Records Interactive Search (CRIS) platform for data of service users in the South London and Maudsley (SLaM) NHS Foundation Trust, tobacco smoking status was retrospectively detected through an algorithm of natural language processing technique, categorising into ‘current smoker’, ‘ex-smoker’ and ‘non-smoker’ by the clinical notes of SMI individuals during 2007–2015. A hierarchical assignment rule was applied following the order of ‘smoker’, ‘ex-smoker’ and then ‘non-smoker’ in an individual. Logistic regression was used to examine the association between smoking and OCS in people with SMI for univariable and multivariable analyses.

**Results:**

We identified 15 479 SMI individuals (56% male; mean age 41 years old), with 90.4% ever smoked. Among them, 2320 (15%) had OCS (without OCD), while 2174 (14%) had a clinical diagnosis of comorbid OCD. After adjusting for demographics and functional status as confounders, both SMI individuals with OCS only and an OCD diagnosis were significantly more likely to have ever smoked (adj. odds ratio 1.47, 95% CI 1.23, 1.76 and adj. odds ratio 1.33, 95% CI 1.11, 1.60, respectively) compared with those without OCD/OCS.

**Conclusions:**

In this large-scale analysis of people with SMI, we found that individuals with OCS or OCD were more likely to have ever smoked.

People with a severe mental illness (SMI), including schizophrenia, schizoaffective disorder (SAD) and bipolar disorder, have a two to three times higher mortality rate than the general population^1^ and reduced life expectancy of 10 to 20 years.^2^ One of the main potential contributors to increased mortality is the high prevalence of tobacco use or smoking among SMI patients.^3,4^ compared with the general population.^5,6^ However, patients with obsessive–compulsive disorder (OCD) have been described as having a lower prevalence of smoking than the general population,^7^ although findings have not been consistent.^8–10^ On the basis of these findings, although related evidence has been accumulating for smoking as an independent risk factor of schizophrenia and bipolar disorder incidence,^11^ the potential interplay between smoking and OCD/OCS in severe mental illness remains unclear.^6^

Obsessive compulsive symptoms (OCS) are usually defined as persistent, intrusive and distressful thoughts (obsessions) and repetitive goal-directed rituals (compulsions).^12^ OCS can occur as a co-morbid condition in schizophrenia, SAD or bipolar disorder. Based on the severity of OCS and the presence of diagnostic criteria, two groups of patients can be described. The first SMI group is sub-syndrome, clinically relevant OCS without having an OCD diagnosis. The second SMI group has received a formal diagnosis of co-morbid OCD and is likely to have more severe OCS.^13^

A growing body of evidence suggests that approximately one quarter to over one third of patients with schizophrenia, bipolar disorder or SAD are found to also display OCS,^14–18^ with over 10% of these SMI patients meeting the full diagnostic criteria of OCD,^18–20^ compared with only two to three percent prevalence of OCD in the general population.^21^ Despite being relatively common in SMI, OCS and OCD have been under-investigated in SMI.^22^

Little is known about tobacco smoking patterns when OCS coexists with SMI. Previous research has predominantly focused on a comorbid diagnosis of OCD and schizophrenia. While one study found people with schizophrenia who smoke to have a lower mean OCS score, compared with people with schizophrenia who did not smoke,^23^ others found no significant difference among patients of schizophrenia with or without OCS,^24–26^ or even higher.^27^ Three further studies observed that the incidences of substance abuse showed mixed results in comparisons between patients of schizophrenia with and without OCD, although these studies did not evaluate nicotine use.^15,28,29^ Older studies have been criticised for having convenience samples, small sample sizes, minimal power and lacking standardised diagnostic criteria for schizophrenia, OCD and OCS.^7,11,25,26^

Through the use of electronic health records, the present study intends to examine the hypothesis that people with SMI, who have experienced OCS or been diagnosed with OCD, have a lower prevalence of smoking compared with those with SMI but without these symptoms in a large sample representing the treated population in a geographic catchment area in South London.

## Method

### Study setting

The data used in this study came from the South London and Maudsley NHS Foundation Trust (SLaM) case register. SLaM provides extensive portfolios of mental health services with a wide range of multidisciplinary and integrated specialist services in southeast London. SLaM is a monopoly service provider for approximately 1.36 million residents over four boroughs (Croydon, Lambeth, Lewisham and Southwark), and accepts severe case referrals from outside of the SLaM catchment area for national tertiary services. Since 2006, all services in SLaM have kept full electronic health records. Currently, the register consists of records for over 500 000 service users, making it the largest electronic case register of its kind in Western Europe. SLaM’s case records provide sources of the SLaM Biomedical Research Centre (SLaM BRC) for secondary data research purposes.

### Clinical records interactive search (CRIS) system

Records were searched and retrieved using SLaM’s CRIS platform. Established in 2008, CRIS allows researchers to access de-identified information from both structured (i.e. coded data) and unstructured (i.e. text fields). The CRIS data resource has been described in detail elsewhere.^30^

### Inclusion criteria

The sample for this analysis comprised patients who had been diagnosed with an SMI before or during the observation window (1 January 2007 to 31 December 2015), recorded in structured fields in accordance with the 10th edition of the World Health Organization (WHO) ICD-10^31^ codes F20, F25 and F31. All included patients were 15 years or older at the time of their first SMI diagnosis before or during the observation window. Those aged over 100 years at initial diagnosis were excluded, as were those with missing age or gender, and included patients were also restricted to those receiving a face-to-face clinical contact at least once within the observation period and to have at least one ascertainment of smoking status in their clinical records.

### Data extraction

Data presented in structured fields in the source record can be retrieved directly via CRIS, supplemented by a range of natural language processing algorithms that are deployed to extract named entities from the large quantities of unstructured (human-authored) texts within the source record,^32,33^ predominantly using Generalised Architecture for Text Engineering (GATE) software.^34^ These include algorithms for recorded diagnosis (Precision: 98%, Recall: 88%), smoking status (Precision: 80%, Recall: 88%), and the OCS app (Precision: 76.9%, Recall: 66.6%).^33^

### Main outcome measure

In the current study, the main outcome measure was tobacco smoking status. Using a previously established natural language processing algorithm,^35^ recorded mentions were categorised into ‘current smoker’, ‘ex-smoker’ and ‘non-smoker’. If the record included more than one mention of different smoking status categories, then smoking status was assigned in a hierarchy where ‘smoker’ took precedence over ‘ex-smoker’ and both ‘smoker’ and ‘ex-smoker’ took precedence over ‘non-smoker’. The data extraction then recoded smoking status as ‘1’, if they had ever smoked (i.e. ‘smoker’ or ‘ex-smoker’) and ‘0’ if they had never smoked (i.e. ‘non-smoker’).

### Exposure variables

The main exposure variable, presence of OCS, was extracted from structured and free-text fields in CRIS, using several approaches. A diagnosis of OCD was taken from both the structured fields (ICD-10 code: F42) and from the unstructured fields, using the diagnosis app. Further data were obtained using the OCS app. The OCS app retrieved instances within the free-text fields where individuals were described as having OCS. This app was also able to distinguish instances in the free-text whereby a patient was described as having a comorbid OCD diagnosis. The development of this app is described in detail in a previous publication.^33^ Data derived from structured fields and the two apps were combined in order to have optimal sensitivity in detecting OCS mentioned within the electronic patient records.^33^ Data from these sources were condensed to produce three exposure categories for each patient: (a) neither OCS nor OCD, (b) OCS without a clinical diagnosis of OCD and (c) OCD, a clinical diagnosis of OCD.

Potential confounding variables, including gender, age (on 1 January 2007 for those with pre-existing SMI diagnoses, or the date of first SMI diagnosis during the observation window), marital status, ethnicity and area level of deprivation, were extracted from the structured fields in CRIS. Marital status was re-classified to in a relationship/cohabiting status. Categories of ‘Cohabiting’, ‘Married’ and ‘Civil Partnership’ were regrouped as ‘1’ (i.e. in a relationship). Categories ‘Divorced’, ‘Divorced/Civil Partnership Dissolved’, ‘Separated’, ‘Single’, ‘Widowed’, ‘Widowed/Surviving Civil Partner’ and ‘Missing’ were coded as ‘0’ (i.e. not in a relationship*)*. Self-reported ethnicity was recoded from 14 self-reported categories into seven modified categories (‘White’, ‘Other White’, ‘South Asian’, ‘East Asian’, ‘Caribbean’, ‘Other Black’ and ‘Mixed or Unknown’). Level of socioeconomic status was assessed based on the patient’s address closest to their first SMI diagnosis and coded according to the Index of Multiple Deprivation (IMD) score at Lower-layer Super Output Area level as defined by the UK Office for National Statistics (ONS). The ONS created the IMD using seven components collated from the national UK census, including ‘income’, ‘employment’, ‘health and disability’, ‘education, skills and training’, ‘barriers to housing and services’, ‘crime’ and ‘living environment’ within an area.^36^ The IMD components are given with specific weightings to reflect the importance of each component. Scores were then split into tertiles: ‘low’ (0–<25.26 percentile), ‘medium’ (25.26–<34.97 percentile), ‘high’ (34.97–100 percentile) and a fourth category assigned to ‘homeless or unknown’. Other variables used for defining our study sample included date of death (if applicable), date of first SMI diagnosis, total contact days within the observation period (number of face-to-face days and number of days in the ward) and the date of the final face-to-face appointment.

Finally, the Health of the Nation Outcome Scale (HoNOS) completed during the observation period was used to evaluate behaviour, symptoms, impairment and domains of social functioning problems, as a routine measure of mental health services in the UK by clinicians for patients’ well-being. This instrument has been well-characterised, including test–retest reliability with intraclass correlation coefficients (ICC) for individual items and total scores, all ranging between 0.74 and 0.88, except the item for aggression (ICC = 0.61), having received ‘good’ to ‘very good’ ICC for the majority of items in the scale in two independent trials, and having performed reasonably well in comparisons with two other scales for equivalent items.^37^ There are 12 items in the HoNOS. Response options follow the format of: ‘0’ (not a problem), ‘1’ (minor problem requiring no action), ‘2’ (mild problem but definitely present), ‘3’ (moderately severe problem) and ‘4’ (severe to very severe problem). In this analysis, the following HoNOS items were included: Hallucinations and delusions, Depressed mood, Over-active aggressive behaviour, Non-Accidental self-injury, Problem drinking or drug taking, Cognitive problems, Physical illness or disability problems, Social relationship problems, Activities of daily living, Standards of living conditions and Occupational and recreational activities. In the case of the present analysis, all HoNOS variables were condensed from five categories into three, due to limited numbers in some categories. The three categories were: ‘0’ (not a problem), ‘1’ (minor problem only) and ‘2–4’ (significant problem).

### Statistical analysis

First, a number of cohort characteristics as well as smoking status were identified and participants were categorised by their OCS-OCD status. Chi-square tests were then carried out to compare across groups for the categorical variables of sociodemographic and clinical factors. Second, logistic regression was performed in order to estimate the crude association between OCS-OCD diagnosis and smoking. A multivariable logistic regression analysis was then undertaken to determine if the association remained, after adjusting potential confounding variables. Finally, the following sensitivity analyses were performed: (a) excluding those whose total face-to-face contact days were less than 30, as those with fewer contact hours may not have had sufficient time for their OCS or smoking status to be recorded, and (b) restricting the sample to include only those who reside within the catchment areas, thereby discarding any serious referrals who were likely to have had unrecorded contact with non-SLaM services. Data analyses were carried out using the statistical software Stata 13 for Windows (StataCorp, College Station, Texas, USA; https://www.stata.com/). The statistical significance level was set at 0.05 (alpha level) for two-tailed tests.

### Ethical considerations

The CRIS data security procedures are presented in detail elsewhere.^38^ Ethical approval was granted for CRIS as a source of data for secondary analysis by the Oxfordshire Research Ethics Committee C (reference: 23/SC/0257) without participants’ informed consent being required for its anonymous essence.

## Results

**Fig. 1:**
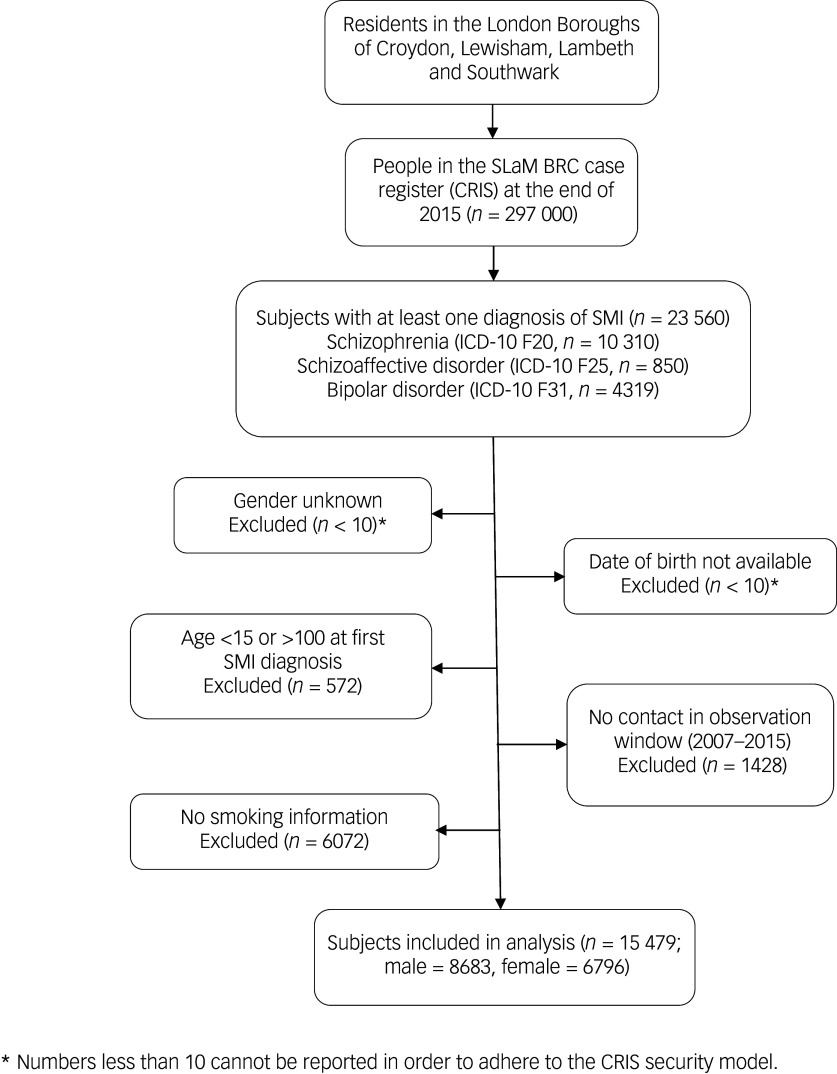
Participant sample flow chart. SLaM BRC, South London and Maudsley Biomedical Research Centre; SMI, severe mental illness.

A total 23 560 patients were identified who had received an SMI diagnosis during the nine-year observation window, of whom 8527 were excluded as detailed in the participant flow chart (Fig. 1). Therefore, the analysed sample comprised 15 479 patients with a mean age of 41.3 (s.d. = 15.6) years old and 6796 (43.9%) of whom were female. Table 1 provides numbers of individuals and smoking status by diagnosis, level of symptom severity and other characteristics. Of the total sample, 13 993 (90.4%) were identified as smokers. The characteristics of individuals by OCS status are shown in Table 2. The majority of them (*n* = 10 985; 71.0%) had no OCD diagnosis nor recorded OCS. Of the remaining individuals, 15.0% (*n* = 2320) were identified as having OCS without a clinical diagnosis of OCD, while 14.0% (*n* = 2174) had an OCD diagnosis. Statistically significant differences between groups were observed for all covariates except gender (Table 2).

**Table 1:** Sample characteristics and percentage of smokers (*N* = 15 479)

Variable	Total	Smokers, *n* (%)
Age^*^ (Mean = 41, s.d. = 15.55, Range: 15–96)		
15–29	4146	3835 (92.50)
30–39	3707	3384 (91.29)
40–49	3601	3301 (91.67)
50–100	4052	3472 (86.26)
Gender^*^		
Female	6796	8691 (86.90)
Male	8683	8086 (93.12)
Ethnicity^*^		
White	6266	5704 (91.03)
White Other	1604	1462 (91.15)
South Asian	386	307 (79.53)
East Asian	458	385 (84.06)
Caribbean	1597	1444 (90.42)
Other Black background	3697	3393 (91.78)
Mixed, other or unknown	1471	1297 (88.17)
Relationship status^*^		
In a relationship (married and cohabited)	13 577	12 435 (91.59)
Not in a relationship (single, divorced and widowed)	1902	1557 (81.86)
Deprivation score in small areas^*^		
Low (1st tertile)	4899	4309 (87.96)
Medium (2nd tertile)	4896	4422 (90.32)
High (3rd tertile)	4916	4531 (92.17)
Homeless or unknown	768	730 (95.05)
Primary psychiatric diagnosis^*^		
Schizophrenia (ICD-10 code F20)	10 310	9472 (91.87)
Schizoaffective disorder (ICD-10 code F25)	850	775 (91.18)
Bipolar disorder (ICD-10 code F31)	4319	3745 (86.71)
Hallucinations and delusions^*^		
Not a problem	5647	5034 (89.14)
Minor problem only	2200	2022 (91.91)
Significant problem	6019	5510 (91.54)
Depressed mood^*^		
Not a problem	5822	5327 (91.50)
Minor problem only	3970	3609 (90.91)
Significant problem	4089	3643 (89.09)
Overactive aggressive behaviour^*^		
Not a problem	7347	6564 (89.34)
Minor problem only	3179	2893 (91.00)
Significant problem	3395	3157 (92.99)
Non-accidental self-injury^*^		
Not a problem	11 695	10 585 (90.51)
Minor problem only	1138	1023 (89.89)
Significant problem	1069	989 (92.52)
Problem drinking or drug taking^*^		
Not a problem	9748	8567 (87.88)
Minor problem only	1438	1373 (95.48)
Significant problem	2611	2559 (98.01)
Cognitive problems		
Not a problem	8193	7383 (90.11)
Minor problem only	3180	2899 (91.16)
Significant problem	2517	2303 (91.50)
Physical illness and disability problems^*^		
Not a problem	8617	871 (91.34)
Minor problem only	2320	2091 (90.13)
Significant problem	2948	2620 (88.87)
Social relationship^*^		
Not a problem	5001	4457 (89.12)
Minor problem only	3670	3335 (90.87)
Significant problem	5157	4733 (91.78)
Activities of daily living^*^		
Not a problem	6354	5674 (89.30)
Minor problem only	3395	3085 (90.87)
Significant problem	4089	3779 (92.42)
Standard of living conditions^*^		
Not a problem	7964	7072 (88.80)
Minor problem only	2596	2402 (92.53)
Significant problem	2917	2745 (94.10)
Occupational and recreational activities^*^		
Not a problem	5898	5272 (89.39)
Minor problem only	3303	3013 (91.22)
Significant problem	4355	4000 (91.85)

* *p*-value <0.05 by a Chi-square test.

**Table 2:** Characteristics of severe mental illness subjects with or without OCS/OCD (*N* = 15 479)

Variables	No OCD or OCS (*n* = 10 985)	OCS (without OCD) (*n* = 2320)	OCD (*n* = 2174)
	*n* (%)		
Age^*^ (Mean = 41.33, s.d. = 15.55, Range: 15–96)			
15–29	2762 (25.14)	558 (24.05)	826 (37.99)
30–39	2663 (24.24)	499 (21.51)	545 (25.07)
40–49	2527 (23.01)	612 (26.38)	462 (21.25)
50–100	3033 (27.61)	651 (28.06)	341 (15.69)
Gender			
Female	4798 (43.68)	1042 (44.91)	956 (43.97)
Male	6187 (56.32)	1278 (55.09)	1218 (56.03)
Ethnicity^*^			
White	4331 (39.43)	907 (39.09)	1028 (47.29)
Other White	1194 (10.87)	211 (9.09)	199 (9.15)
South Asian	288 (2.62)	44 (1.90)	54 (2.48)
East Asian	334 (3.04)	64 (2.76)	60 (2.76)
Caribbean	1101 (10.02)	321 (13.84)	175 (8.05)
Other Black background	2637 (24.00)	601 (25.91)	459 (21.11)
Mixed, other or unknown	1100 (10.01)	172 (7.41)	199 (9.15)
Relationship status^*^			
In a relationship	9502 (86.50)	2125 (91.59)	1950 (89.70)
Not in a relationship	1483 (13.50)	195 (8.41)	224 (10.30)
Deprivation score in small areas^*^			
Low (1st tertile)	3453 (31.43)	700 (30.17)	746 (34.31)
Medium (2nd tertile)	3500 (31.87)	718 (30.95)	678 (31.19)
High (3rd tertile)	3448 (31.39)	800 (34.48)	668 (30.73)
Homeless	548 (5.32)	102 (4.40)	82 (3.77)
Primary psychiatric diagnosis			
Schizophrenia (ICD-10 code F20)	7293 (66.39)	1604 (69.14)	1413 (65.00)
Schizoaffective disorder (ICD-10 code F25)	595 (5.42)	134 (5.78)	121 (5.57)
Bipolar disorder (ICD-10 code F31)	3,097 (28.19)	582 (25.09)	640 (29.44)
Hallucinations and delusions^*^			
Not a problem	3993 (41.00)	817 (37.79)	837 (42.60)
Minor problem only	1525 (15.66)	348 (16.10)	327 (16.64)
Significant problem	4221 (43.34)	997 (46.11)	801 (40.76)
Depressed mood^*^			
Not a problem	4110 (42.17)	950 (43.86)	762 (38.70)
Minor problem only	2834 (29.08)	619 (28.58)	517 (26.26)
Significant problem	2802 (28.75)	597 (27.56)	690 (35.04)
Overactive aggressive behaviour^*^			
Not a problem	5234 (53.54)	1101 (50.71)	1012 (51.27)
Minor problem only	2201 (22.51)	520 (23.95)	458 (23.20)
Significant problem	2341 (23.95)	550 (25.33)	504 (25.53)
Non-accidental self-injury^*^			
Not a problem	8256 (84.61)	1839 (84.79)	1600 (81.01)
Minor problem only	779 (7.98)	154 (7.10)	205 (10.38)
Significant problem	723 (7.41)	176 (8.11)	170 (8.61)
Problem drinking or drug taking^*^			
Not a problem	6877 (70.95)	1534 (71.42)	1337 (68.35)
Minor problem only	997 (10.29)	219 (10.20)	222 (11.35)
Significant problem	1819 (18.77)	395 (18.39)	397 (20.30)
Cognitive problems^*^			
Not a problem	5846 (59.92)	1148 (53.07)	1199 (60.83)
Minor problem only	2195 (22.50)	563 (26.03)	422 (21.41)
Significant problem	1715 (17.58)	452 (20.90	350 (17.76)
Physical illness and disability problems^*^			
Not a problem	6060 (62.14)	1240 (57.33)	1317 (66.85)
Minor problem only	1587 (16.27)	417 (19.28)	316 916.04)
Significant problem	2105 (21.59)	506 (23.39)	337 (17.11)
Social relationship^*^			
Not a problem	3719 (38.31)	644 (29.84)	638 (32.50)
Minor problem only	2606 (26.85)	572 (26.51)	492 (25.06)
Significant problem	3382 (34.84)	942 (43.65)	833 (42.44)
Activities of daily living^*^			
Not a problem	4644 (47.78)	817 (37.89)	893 (45.49)
Minor problem only	2391 (24.60)	535 (24.81)	469 (23.89)
Significant problem	2684 (27.62)	804 (37.29)	601 (30.62)
Standard of living conditions^*^			
Not a problem	5741 (60.70)	1101 (52.45)	1122 (58.44)
Minor problem only	1771 (18.72)	446 (21.25)	379 (19.74)
Significant problem	1946 (20.58)	552 (26.30)	419 921.82)
Occupational and recreational activities^*^			
Not a problem	4195 (44.05)	886 (42.13)	817 (42.33)
Minor problem only	2358 (24.79)	505 (24.01)	440 (22.80)
Significant problem	2970 (31.19)	712 (33.86)	673 (34.87)

OCS, obsessive–compulsive symptoms; OCD, obsessive–compulsive disorder.

* *p*-value <0.05 by a Chi-square test.

Table 3 summarises univariable and multivariable analyses of associations between exposures of interest (OCS/OCD status) and smoking considering various adjustments in blocks of potential confounders. A likelihood ratio test indicated that it was appropriate to use age as a continuous variable within the models. The results show a significant relationship between having experienced OCS (without a clinical diagnosis of OCD) and having ever smoked in individuals with SMI across all models, including in the fully adjusted model. In the univariable analysis, people with SMI with OCS but without a clinical diagnosis of OCD (odds ratio 1.62, 95% CI 1.36, 1.92, *p* < 0.01), and SMI individuals with a clinical diagnosis of OCD (odds ratio 1.50, 95% CI 1.26–1.78, *p* < 0.01) were more likely to have ever smoked compared to individuals with SMI but neither OCS nor OCD. Both of these relationships remained statistically significant in the fully adjusted model (odds ratio 1.47, 95% CI 1.23, 1.76, *p* < 0.01 and odds ratio 1.33, 95% CI 1.11, 1.60, *p* < 0.01).

**Table 3:** Univariable and multivariable analysis on the relative risk of smoking for OCS/OCD status in individuals with schizophrenia, bipolar disorder or schizoaffective disorder (*N* = 15 479)

OCS/OCD status	Crude	Adjusted for age and gender	Adjusted for demographics^a^	Adjusted for SMI diagnosis^b^	Adjusted for severity^c^	Adjusted for other physical and mental health^d^	Adjusted for functional status^e^	Fully adjusted^f^
	Odds ratio (95% CI)							
No OCD or OCS	Ref.	Ref.	Ref.	Ref.	Ref.	Ref.	Ref.	Ref.
OCS (without a clinical diagnosis of OCD)	1.62 (1.36, 1.92)^*^	1.64 (1.37, 1.94)^*^	1.55 (1.30, 1.84)^*^	1.59 (1.34, 1.89)^*^	1.58 (1.33, 1.87)^*^	1.63 (1.37, 1.94)^*^	1.52 (1.28, 1.81)^*^	1.47 (1.23, 1.76)^*^
OCD (with a clinical diagnosis of OCD)	1.50 (1.26, 1.78)^*^	1.35 (1.13, 1.61)^*^	1.34 (1.12, 1.60)^*^	1.51 (1.27, 1.80)^*^	1.51 (1.27, 1.80)^*^	1.46 (1.23, 1.74)^*^	1.47 (1.24, 1.75)^*^	1.33 (1.11, 1.60)^*^

OCS, obsessive–compulsive symptoms; OCD, obsessive–compulsive disorder; SMI, severe mental illness; Ref., reference.

* *p*-value <0.05.

a. Age, gender, ethnicity, relationship, and deprivation score.

b. Priority diagnosis (schizophrenia > bipolar disorder > schizoaffective).

c. Overactive aggressive behaviour, Hallucinations and delusions, and Depressed mood.

d. Non-accidental self-injury, Problem drinking or drug taking, Physical illness and disability problems.

e. Impairment in activities of daily living, Standard of living conditions, Occupational and recreational activities and Social relationships.

f. Adjusted for all of the above variables.

Table 4 displays the results from two sets of the sensitivity analyses. First, some service users may not have had sufficient contact to identify OCS or to correctly identify smoking status. Therefore, any service users who had less than 30 clinical contact days within the observation period were excluded. After this, the association between smoking and OCS (without a clinical diagnosis of OCD) remained significant for SMI patients (odds ratio 1.53, 95% CI 1.23, 1.91, *p* < 0.01). However, the association between smoking and OCD (with a clinical diagnosis of OCD) was no longer statistically significant (odds ratio 1.18, 95% CI 0.96, 1.47, *p* = 0.12). In the second sensitivity analysis, we removed all patients who were referred from outside the SLaM catchment area. These people are more likely to be more severe cases referred to SLAM for specialised secondary or tertiary mental health care. In the remaining patients from the boroughs in the SLAM catchment area, the effect estimate for the fully adjusted association between OCS (without a clinical diagnosis of OCD) and smoking was slightly stronger (odds ratio 1.61, 95% CI 1.32, 1.96, *p* < 0.01), whereas the fully adjusted effect estimate for the association between OCD (with a clinical diagnosis of OCD) and smoking remained comparable (odds ratio 1.33, 95% CI 1.10, 1.62, *p* < 0.01).

**Table 4:** Sensitivity analyses using the fully adjusted model

OCS/OCD status	Only include if they have more than 30 days of contact (*n* = 11 799)^a^	Only include those who live within the SLAM catchment area (*n* = 12 494)^a^
	Odds ratio (95% CI)	
No OCD or OCS	Ref.	Ref.
OCS (without a clinical diagnosis of OCD)	1.53 (1.23, 1.91)^*^	1.61 (1.32, 1.96)^*^
OCD (with a clinical diagnosis of OCD)	1.18 (0.96, 1.47)	1.33 (1.10, 1.62)^*^

OCS, obsessive–compulsive symptoms, OCD, obsessive–compulsive disorder; SLAM, South London and Maudsley; Ref., reference.

* *p*-value <0.05.

a. Adjusted for age, gender, ethnicity, relationship, deprivation score, priority diagnosis (schizophrenia > bipolar disorder > schizoaffective), Overactive aggressive behaviour, Hallucinations and delusions, Depressed mood, Non-accidental self-injury, Problem drinking or drug taking, Physical illness and disability problems, Impairment in activities of daily living, Standard of living conditions, Occupational and recreational activities and Social relationships.

## Discussion

### Main findings and interpretation

In the current study, we found that individuals with SMI were more likely to have ever smoked, based on electronic recording in their case notes, if they displayed OCS (with or without a clinical diagnosis of OCD), compared with those with SMI who did not have OCS. In addition, this association remained significant after adjusting for a broad range of potential confounding variables.

### Comparisons of previously published studies

These findings contradict most of the current literature on this topic. Only four previous studies have investigated the comorbidity of SMI and OCD in its relationship with smoking. Of these, de Haan et al revealed a relationship in the opposite direction, reporting smoking as significantly lower in comorbid schizophrenia and OCD patients compared with those with schizophrenia alone.^15^ Dome et al reported a null finding, with OCS not remarkably related with smoking habits among SMI patients.^25^ Similarly, Fawzi et al found that the smoking prevalence among patients with both schizophrenia and OCS was 85.3%, not significantly different from the 90.6% prevalence among patients without OCS.^26^ Also, Dekker et al found no statistically significant differences in smoking between three groups of schizophrenia patients according to the severity of OCS in a cohort study (*N* = 1005).^24^ However, in an Australian study of people with psychosis, Bosanac et al reported that those with OCS had a similarly raised relative risk of smoking in the past 12 months to the results we report here.^27^ Contrary to our hypothesis, we did not observe that OCS reduced the likelihood of smoking as had been reported previously.^15^ These mixing findings may reflect the incompletely addressed issues of different study settings, definitions of the study individuals and the methodology of smoking status detection under the limitation of smaller sample sizes of these related studies.

### Strengths and limitations

A major strength of this study is the large sample size, the largest investigation of this research question to date, to the best of our knowledge. Repeatedly, previous studies have been criticised for involving small samples,^8^ resulting in insufficient statistical power^25^ and limited generalisability.^15^ In addition, many studies have recruited participants using convenience sampling procedures, frequently resulting in low response rates,^11^ potential selection bias^26^ and high dropout rates.^24^ In contrast, using electronic mental health care records, the current study included within a cohort virtually all patients who had been in contact with specialists in mental health services within a defined geographic catchment area for over nine years of observation. The data in this study should be broadly representative of patients from other culturally diverse urban and suburban areas, in comparison with several previous studies involving predominantly Caucasian females from similar social backgrounds.^8^ The comprehensive data source also allowed a wide range of potential confounders to be considered. In addition, application of natural language processing algorithms, a unique feature of this study, enhanced the ability to distinguish between patients with OCS with and without a clinical diagnosis of OCD.

Nonetheless, there were still several limitations to be borne in mind. We adopted secondary data which were routinely collected for clinical purposes rather than research. Moreover, despite controlling for numerous potential confounders, there may still be residual confounding. Information regarding the duration of the illness as well as the distinct symptom dimensions of their OCS and more specifically quantified features of their smoking habits (e.g. number of cigarettes per day and duration of smoking) would have been valuable in the analysis, but was not available. In addition, individuals categorised as ‘smoking ever’ may also include individuals who started smoking but stopped shortly after. We were unable to distinguish this from these data. Additionally, only limited data were available prior to 2006. This study was carried out in one single catchment area in a high-income country, potentially resulting in limited generalisability across countries. Meanwhile, although the natural language processing algorithms used were innovative and performed satisfactorily, they were not perfect, leaving potential misread, misclassified or unidentified information. Finally, this was a cross-sectional analysis which identified a correlation between OCS/OCD and smoking in patients with SMI, but did not permit the direction of causal relationship to be inferred. It is possible that OCS had an impact on smoking or that those who smoked were more likely to exhibit OCS or have their OCS symptoms detected by a clinician. In this investigation, the association between OCD and having ever smoked was no longer significant after removing those with less than 30 days of clinical contact in the sensitivity analysis. It may reflect higher rates of smoking in SMI patients with comorbid OCD who have had less clinical contact or it may be indicative of misclassification where those with less clinical contact are less likely to be classified correctly regarding their smoking status. However, although the strength of this association was reduced and no longer significant, it remained in the same direction.

### Implications and future directions

While there have been several studies investigating smoking in relation to OCD and SMI as distinct disorders, research is sparse on patients with a comorbidity of these two disorders and/or underlying symptom profiles. The present study provides a novel finding with potential implications for clinical practice and public health about treatment and management plans. These findings have the potential to help understand the mechanisms of smoking and SMI, as well as to contribute to the underlying relationship between OCS, SMI and smoking. Ultimately, this study has identified a subgroup at an increased risk of tobacco smoking and thus requires particular attention.

Following the excessive smoking among patients with SMI identified in previous research, smoking cessation interventions have been developed to meet the needs of individuals with SMI and such applications have been recognised.^39,40^ On the contrary, due to the low prevalence of smoking in patients with OCD, interventions for OCD rarely focus on smoking cessation. In addition, smoking behaviour may vary depending on whether patients have both OCD and SMI or not, and may thus impact their treatment targeting smoking cessation or reduction. It is unclear how or why smoking behaviour may differ between these groups of patients or what kind of interventions may be more effective for them. However, as implied by these findings, clinicians can expect that when patients present with both an SMI and OCS, they are likely to smoke even more than if they had either alone. For that reason, a focus on smoking cessation interventions should be given increased attention by clinicians as part of a comprehensive treatment plan. Future research would benefit from inferring causation from the correlation found, as well as including more information regarding patients’ smoking behaviour.

In conclusion, people with OCS and/or OCD in addition to SMI are more likely to have ever smoked compared with individuals with SMI but no OCS or OCD. This is the largest study of this question to date. Nevertheless, additional research is necessary to further explore these findings and clarify underlying mechanisms.

## Data Availability

Data that support the findings of this study are available from the corresponding author, C.-K.C., upon reasonable request. Nonetheless, the data can only be accessed by specifically permitted researchers within a secured firewall, in a similar manner as the authors.
